# The Joint Interagency Task Force and the Global Steering Committee for the Quality Assurance of Health Products: Two New and Proactive Approaches Promoting Access to Safe and Effective Medicines

**DOI:** 10.4269/ajtmh.15-0208

**Published:** 2015-06-03

**Authors:** Martin Cinnamond, Tom Woods

**Affiliations:** Joint Interagency Task Force (JIATF), The Global Fund to Fight AIDS, Tuberculosis and Malaria, Geneva, Switzerland

## Introduction

Financing mechanisms such as the Global Fund to Fight AIDS, Tuberculosis and Malaria^1^ support programs run by local experts in countries and communities that most need funding. Those programs help to save lives every day. However, expanding access to medicines on a global scale can face challenges posed by falsified, substandard, stolen, and diverted medicines. The Joint Interagency Task Force (JIATF) is an initiative that offers a new, proactive, and intelligence-led approach to safeguarding the delivery of quality medicines for major donor organizations and protecting public health by identifying falsified medicines in countries where they appear. By also focusing on the issue of stolen and diverted medicines, JIATF provides an important assurance mechanism. A core focus area of the Global Fund component of JIATF (GF-JIATF) is its National Engagement Strategy (NES), which serves as a launch pad to develop partnerships and provide training and logistical support to partners within a country's national drug regulatory and law enforcement community. This national system strengthening compliments and augments health systems strengthening overall. Through its work, GF-JIATF is assisting its drug regulatory agency and law enforcement partners at the national level to identify vendors engaged in the trade of illicit medicines, providing support to successful enforcement operations and helping to disrupt those criminal networks engaged in this trade. These outputs promote better public health outcomes by removing falsified products from circulation. Through a carefully developed framework of data collection and analysis, its NES, and the establishment of a broad coalition of international partnerships, the GF and its fellow core members in JIATF are helping to protect drug quality as an integral part of their organization's core mission to expand access to safe and effective medicines.

The GF is also a founding member and the Secretariat for the Global Steering Committee (GSC) for quality assurance of health products, which is a voluntary coalition of health development institutions focused on improved access to safe and effective medicines and chaired by Norbert Hauser, the chair of the GF Board. Current Core membership of GSC includes Gavi, the Global Fund, the United Nations Development Program (UNDP), UNITAID, the U.S. Presidents Malaria Initiative (PMI), the U.S. Agency for International Development (USAID), the U.S. Food and Drug Agency, the World Bank, and the World Health Organization (WHO). Inaugurated in November 2014, the GSC will harness the collective efforts of multilateral and bilateral organizations, national authorities, nongovernmental organizations (NGOs), and drug companies to facilitate an enhanced assurance framework for both quality and supply chain integrity of health products.

## JIATF Mission and Membership

The mission of JIATF is to prevent, detect, and respond to theft, diversion, falsification, and substandard quality GF- and USAID-funded medicines.^2^ Although the GF had been collaborating with USAID in relation to these issues on a reactive and ad hoc basis for a number of years, the GF developed the concept of establishing a more formalized JIATF in 2012 recognizing that a more comprehensive, proactive, and intelligence-led approach was needed to better tackle the issues. JIATF comprises personnel from a dedicated team within the GF, USAID (from PMI^3^ and its Office of Inspector General^4^ and in 2014, the UNDP^5^ under the auspices of its Office of Audit and Investigations joined the Task Force.^6^ This article focuses largely on the GF-JIATF. During the first 2 years of the initiative, GF-JIATF initially focused its efforts on artemisinin-based combination therapy (ACT) medicines. However, following the success of the approach, the GF-JIATF is expanding its focus in 2015 into other essential medicines, including tuberculosis treatments.

Within the GF, JIATF's proactive operations and planning expand the organization's ability to move from a reactive, ad hoc approach on falsified, substandard, stolen, and diverted medicines to a much more forward-looking and risk-based capacity. GF-JIATF helps the GF understand the pervasive threat of stolen, diverted, falsified, and substandard medicines. It also enables the GF to respond in more targeted and effective manner in those countries where problems are identified through GF-JIATF's activities. This innovative work offers a broader intelligence picture and is contributing to the identification of newly discovered falsified antimalarial medicines, including WHO-prequalified generic manufactured products.^7^

## GF-JIATF's Strategic Framework

GF-JIATF's strategic framework comprises three foundational pillars: data gathering and analysis, an NES, and an International Engagement Strategy (IES).

### Data gathering and analysis.

To be able to properly respond to the challenges of stolen, diverted, falsified and substandard medicines, it is critical that a level of ground truth is established as to the scope and scale of the problem. Through a variety of sources of information and activities JIATF is developing such a picture in over a dozen countries in sub-Saharan Africa and, in 2014, expanded its focus to also include southeast Asia. In terms of falsified ACTs, GF-JIATF is proactively identifying illicit products beyond its own supply chains. Under the Affordable Medicines Facility-malaria program,^8^ the GF has broadened access to quality antimalarial medicines via a subsidized program whereby ACTs bearing a Green Leaf logo are sold throughout the commercial sector in several countries. Sadly, like many quality brands, organized criminal networks have actively targeted these products, cynically preying on vulnerable patients who recognize that the Green Leaf logo on the authentic products represents a mark of quality and trust. Forensic analysis conducted by GF-JIATF in collaboration with its partners, U.S. Centers for Disease Control and Prevention (CDC) and Georgia Institute of Technology, confirms that these falsified products contain little or no active pharmaceutical ingredient posing an acute public health risk to individuals who have contracted malaria and are in need of remedial therapeutic attention.

This public health risk is the driving motivation behind GF-JIATF seeking out and identifying these illicit products within the commercial sector. Not only do these activities help to remove dangerous falsified products from public circulation, but they also serve to ensure that the important trust, which has been painstakingly developed in respect of the quality, for authentic ACTs bearing the Green Leaf logo is maintained. GF-JIATF is developing an increasingly detailed picture of hot spots where falsified ACTs are circulating in sub-Saharan Africa. This information is disseminated to a multitude of partners, including WHO Substandard, Spurious, Falsely-labeled, Falsified, and Counterfeit Surveillance and Monitoring Unit within the Safety and Vigilance team.^9^ GF-JIATF collaborates closely with the Surveillance and Monitoring Unit, sharing its findings of falsified ACTs, which have informed several WHO Drug Alerts focusing on both generic and innovator manufactured products.^7^ GF-JIATF also shares its finding with the National Medical Regulatory Authorities (NMRAs) and law enforcement agencies partners under the NES where it is then used to inform targeted national enforcement operations against those involved in the trade of illicit medicines. Moving forward, GF-JIATF further disseminates this public health information through enhanced awareness raising initiatives conducted in collaboration with GF country teams and implementers of GF grants.

GF-JIATF also works closely with pharmaceutical manufacturing industry, maintaining strong collaborative partnerships with both the generic (e.g., Cipla and Ipca) and innovator (e.g., Novartis and Sanofi) companies. GF-JIATF's findings provide ample evidence that both sections within the industry are at risk of their products being falsified by criminal networks. GF-JIATF has identified falsified versions of both generic manufacturer ACTs as well as innovator manufacturer ACTs both bearing the GF-promoted Green Leaf logo on the packaging. These falsified products were proactively identified via GF-JIATF's use of rapid authentication technology. A handheld TruScan RM Analyzer device (Thermo Fisher Scientific, Waltham, MA)^10^ enabled GF-JIATF to screen those products acquired during the course of their activities. Products flagged as suspect, together with all products bearing batch numbers, which have been confirmed by the WHO Drug Alert system as falsified, are sent on for comprehensive laboratory testing at GF-JIATF partners, the U.S. CDC and Georgia Institute of Technology.

### National engagement strategy.

Perhaps most critical to JIATF's sustained and long-term success is its partnership with NMRAs and national law enforcement agencies. GF-JIATF's NES provides essential local knowledge and support for near-term enforcement efforts. Long-term strategies simply must rely on a NES that invests in country-level ownership, builds capacity where needed, and results in an expanding network of capable and trusted partnerships. Whereas the mission is saving lives through access to safe and effective medicines, GF-JIATF looks well beyond its immediate and limited goals of protecting only donor supply chains. National system strengthening builds on a foundation of shared interests that ultimately supports the broader health goals of a country.

Pharmaceutical crime is a complex activity that often spans cross-border networks and employs sophisticated, agile, and well-funded strategies. To help individual countries manage the threat, GF-JIATF is engaging in train-and-equip support programs, helping drug regulatory and law enforcement entities enhance their investigative capacities so that prosecutions of pharmaceutical crime can become more likely and lead to successful convictions. Since 2013, GF-JIATF has been partnering with U.K. Police to provide specialist intelligence analysis training to NMRA enforcement teams and other law enforcement agencies and in early 2015 commenced the provision of similar training for Francophone west African NES partners. This training is then augmented with the provision of specialized computer software and hardware. GF-JIATF is now considering measures that expand deployment of authentication and testing devices to its NMRAs and law enforcement partners. Rapid and mobile such handheld analytical devices as the TruScan have been used for positive effect in Nigeria since 2010.^11^ The devices help drug regulators and supply chain managers make accurate and quick field-based determinations on the authenticity and even quality of medicines, which can act as a catalyst for seizures and removing hazardous or ineffective products from public access.

In September 2014, GF-JIATF expanded its training program, delivering a specialist program focusing on effectively investigating pharma crime. Hosted at Tanzania's Prevention and Combating of Corruption Bureau, the 3-day event brought together representatives from the NMRAs, police, anticorruption bureaus, financial intelligence units, and Department of Public Prosecutions from Malawi, Tanzania, and Zambia. Not only do these trainings bolster capabilities, but also they provide ideal forums for different agencies from different countries to come together, develop rapport, and build working relationships. With pharma crime often containing cross-jurisdictional elements, such opportunities to forge these partnerships is a critical component in providing an appropriate response.

A good example of how GF-JIATF's activities and data collection lead the way to proactive enforcement measures took place in Togo in May 2014. Together with Interpol and national authorities, the operation was directly shaped by GF-JIATF's detailed targeted intelligence. The ensuing enforcement activities resulted in the seizure of thousands of falsified ACTs, which yielded an immediate public health benefit by removing the illicit products from the market place.^12^ By deploying on the ground in support of national authorities, GF-JIATF is also able to provide the necessary operational guidance to promote follow-up investigations focused on criminal networks that support the importation and distribution of illicit pharmaceuticals.

Further GF-JIATF support to national enforcement operations are currently being planned and will take place during 2015. GF-JIATF also plans to further leverage coordination and cooperation through regionally focused initiatives including the West African Health Organization.

### International engagement strategy.

Although NES forms the bedrock for current and future GF-JIATF efforts to counter falsified, substandard, stolen, and diverted medicines, the very nature of the threat makes an IES an essential element. As GF-JIATF expands the coalition of the willing from within the large-scale donor agency community, it will also rely increasingly on both public and private sector international partners with global reach and specific technical capacity. Another key partner for GF-JIATF under the IES is Interpol, which has been heavily engaged in fighting pharma crime for many years. Interpol has been instrumental in supporting GF-JIATF develop its NES and has also facilitated GF-JIATF personnel to deploy during its major multi-country pharma crime operations. Joint Interpol/GF-JIATF training initiatives have also been delivered in Benin, Malawi, and Zambia to NMRA and law enforcement personnel since 2013.

## Broadening the Focus: From Downstream to Upstream

The complexity of the global pharmaceutical crime threat also dictates that the GF and other large donor agencies make efforts to protect the integrity of medicines. As the NES strategy promotes “downstream” visibility and security improvements, GF-JIATF will expand its cooperation with the manufacturers of both generic and branded medicines to improve “upstream” visibility and security. Assurance of drug quality, together with efforts to detect falsified medicines, promotes stronger public health outcomes. GF-JIATF will expand its current focus via collaboration with specialist entities to enable good manufacturing process audits of suppliers, thereby strengthening an end-to-end quality assurance approach to all medicines procured and funded by the GF. The large scope, scale, and geographic distribution of pharmaceutical manufacturers make drug quality assurance a challenging priority. Although national and subregional track and trace systems emerge, the systems lack an interoperability component that would reliably provide the “book ended” type of security that large donor organizations might use to enhance public safety. Therefore, GF-JIATF is actively exploring opportunities to test and scale solutions that make the best use of track- and trace-oriented packaging. This work again points to a critical need to expand public and private partnerships at the international level.

## JIATF Support for the GSC for Quality Assurance of Health Products

JIATF relies on an expanding IES and its work informs a broader global coalition effort. The GSC for quality assurance of health products will harness the collective efforts of multilateral and bilateral organizations, regional authorities, NGOs, manufacturers, and technical partners to facilitate an enhanced framework for both quality and supply chain integrity of medicines and health products. The GF is supporting a Secretariat to ensure day-to-day administrative and technical support of the GSC., Norbert Hauser, the former inspector general of the GF, was appointed to serve as the GSC's first chairman during the inaugural meeting convened in Geneva, Switzerland, in November 2014.

Many of the individual GSC core are already heavily involved in several broad activities focusing on improving access to safe medicines. However, there is an increasing recognition that outcomes can be further improved through enhanced coordination and the development of a more joined-up approach. Meeting on a quarterly basis, the GSC is a voluntary coalition and its initial members represent organizations that fund and/or procure life-saving medicines or provide technical expertise associated with efforts to combat falsified and substandard medicines. GF-JIATF's ongoing NES as well as its data gathering and analysis will provide an important contribution to the GSC's focus and cooperation framework. The GSC has established five working groups based on a consensus view of areas where the combined work of its members can make unique contributions: 1) supporting NMRA, drug quality and supply chain authority; 2) data gathering, reporting, sharing, and analysis; 3) information dissemination and public awareness; 4) enforcement; and 5) financing of GSC initiatives. Participants in working groups include GSC core members, together with specialist regional entities, NGOs, private sector representatives, and any other entities that may benefit GSC outcomes. Membership of the working groups is to be endorsed periodically by the GSC core members.

## Conclusion

Together, JIATF and the GSC represent important progress for the international donor agency community in respect of addressing the challenges of stolen, diverted, falsified, and substandard medicines, within the wider ambit of providing greater access to safe medicines. The combined capacities of partners make coordination a critical asset to promote access to safe and effective medicines. And the combined challenges faced by partners make global cooperation essential, as the public health threat posed by falsified and substandard medicines has never loomed larger. Solutions cannot hope to be achieved through unilateral actions, and it is only by working together that further success can be achieved.
